# Profiling the dynamic expression of checkpoint molecules on cytokine-induced killer cells from non-small-cell lung cancer patients

**DOI:** 10.18632/oncotarget.9871

**Published:** 2016-06-07

**Authors:** Lin Zhang, Jian Wang, Feng Wei, Kaiyuan Wang, Qian Sun, Fan Yang, Hao Jin, Yu Zheng, Hua Zhao, Limei Wang, Wenwen Yu, Xiying Zhang, Yang An, Lili Yang, Xinwei Zhang, Xiubao Ren

**Affiliations:** ^1^ Department of Immunology, Tianjin Medical University Cancer Institute and Hospital, Tiyuanbei, Tianjin, China; ^2^ Department of Biotherapy, Tianjin Medical University Cancer Institute and Hospital, Tianjin, China; ^3^ National Clinical Research Center of Cancer, Tianjin, China; ^4^ Key Laboratory of Cancer Immunology and Biotherapy, Tianjin, China

**Keywords:** checkpoint molecule, cytokine-induced killer cell (CIK), non-small-cell lung cancer (NSCLC), immunotherapy

## Abstract

Immune checkpoints associate with dysfunctional T cells, which have a reduced ability to clear pathogens or cancer cells. T-cell checkpoint blockade may improve patient survival. However, checkpoint molecules on cytokine-induced killer (CIK) cell, a non-specific adoptive immunotherapy, remain unknown. In present study, we detected the dynamic expression of eight major checkpoint molecules (CTLA-4, PD-1, PD-L1, TIM-3, CEACAM-1, LAG-3, TIGIT and BTLA) on CIK cells from NSCLC patients. The majority of these molecules, except BTLA, were sharply elevated during the early stage of CIK cell culture. Thereafter, PD-1 and TIGIT expressions decreased gradually towards the initial level (day 0). Moreover, CTLA-4 faded away during the later stage of CIK culture. LAG-3 expression decreased but was still significantly higher than the initial level. Of note, PD-L1 remained stably upregulated during CIK culture compared with PD-1, indicating that PD-L1 might act as an inhibitory molecule on CIK cells instead of PD-1. Furthermore, TIM-3 and CEACAM1 were strongly expressed simultaneously during long-term CIK culture and showed a significant and mutually positive correlation. BTLA displayed a distinct pattern, and its expression gradually decreased throughout the CIK culture. These observations suggested that CIK cells might be partly exhausted before clinical transfusion, characterized by the high expression of PD-L1, LAG-3, TIM-3, and CEACAM-1 and the low expression of TIGIT, BTLA, PD-1, and CTLA-4 compared with initial culture. Our results imply that implementing combined treatment on CIK cells before transfusion via antibodies targeting PD-L1, LAG-3, TIM-3, and CEACAM-1 might improve the efficiency of CIK therapy for NSCLC patients.

## INTRODUCTION

T cells have important roles in anti-tumor and antiviral immune responses. The appropriate activation of antigen-specific T cells leads to their clonal expansion and effector function (e.g., attacking target cells by cytotoxic T lymphocytes (CTLs)) [1]. However, in the setting of malignancy, multiple mechanisms of immune suppression exist, such as IDO, IL-10, Treg, and myeloid-derived suppressor cells (MDSCs), thus preventing effective anti-tumor immunity [2–4].

Another essential mechanism of immune resistance is attributed to checkpoint regulators, which refer to a plethora of inhibitory pathways in the immune system that inhibits the development or function of killer and pro-inflammatory lymphocytes [5]. Tumors co-opt certain immune checkpoint pathways as a major mechanism to avoid immune attacks, particularly against T cells, which are specific for tumor antigens [5]. These negative receptors included cytotoxic T lymphocyte antigen 4 (CTLA4), programmed death protein 1 (PD- 1), lymphocyte activation gene 3 protein (LAG3), T-cell immunoglobulin domain and mucin domain 3 (TIM3), T-cell immunoreceptor with Ig and ITIM domains (TIGIT), and B and T lymphocyte attenuator (BTLA), et al. [1].

The two major checkpoints, CTLA-4 and PD-1, have been actively studied, and the blockade of these pathways via their antibodies demonstrated unprecedented durable response in melanoma patients. These antibodies, including ipilimumab (anti-CTLA-4 antibody), pembrolizumab, and nivolumab (anti-PD-1 antibody), have achieved US Food and Drug Administration (FDA) approval successively [6, 7]. Some of the new checkpoint inhibitors, such as antibodies targeting LAG-3 or TIM- 3, are close to clinical development, particularly in combination with PD-1 inhibitors [8].

Although the checkpoint regulators on dysfunctional T cells, which have limited ability to effectively eliminate tumors, have gained considerable attention, these inhibitory receptors on cytokine-induced killer (CIK) cells remain neglected. CIK cells, which were first reported by Schmidt Wolf in 1990s, have been recognized as another candidate approach for adoptive cell therapy against tumors [9]. Heterogeneous CIK cells consist of two major populations, CD3+CD56- and CD3+CD56+ cells. The CD3+CD56+ cells (also called NKT cells), which have been considered as the most potent cytolytic subset, have the characteristics of NK and T cells [10, 11]. In addition, CIK cells have a broad spectrum of targeted tumors [9, 12]. At present, CIK therapies have been applied for almost all types of solid tumors and hematological malignancies all over the world [13]. In our previous study, we demonstrated that CIK cell immunotherapy could improve the prognosis of non-small-cell lung cancer (NSCLC), which is the leading cause of cancer deaths in many advanced countries [14, 15]. Although CIK cell therapy is effective and has been widely applied, this type of therapy is only suitable for approximately 30%–40% patients and has a variable effect among patients. Furthermore, the underlying mechanism of CIK cell therapy is still unclear. Thus, the current study aims to evaluate the dynamic expression of checkpoint regulators during the proliferation of CIK cells derived from NSCLC patients, and also aims to provide the key inhibitory molecules that could affect the effectiveness of CIK therapy.

## RESULTS

### Subsets of CIK cells in small-scale culture system

**Figure 1:**
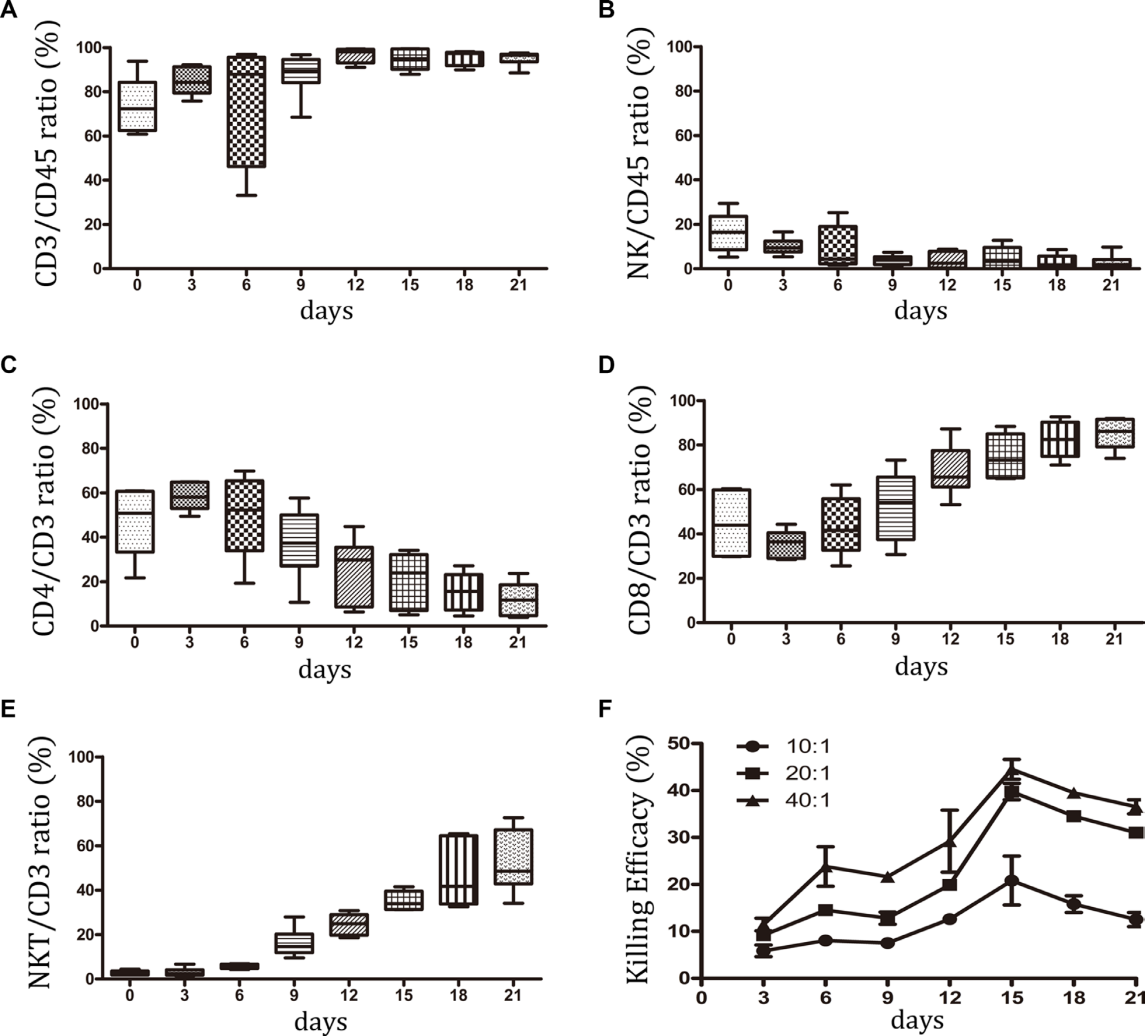
Subsets of CIK cells were detected in small-scale culture system PBMCs from six patients with NCSLS were selected for small-scale CIK culture. During 21 days of incubation, the subsets of CIK cells were studied by FCM analysis. (**A–B**) The dynamic percentage of CD3+ and CD3-CD56+ NK cells in CD45+ lymphocyte were shown. (**C–E**) Among the CD3+ cells, the percentage of CD3+CD4+ T, CD3+CD8+ T, CD3+CD56+ NKT cells were detected during the culture. (**F**) The dynamic assay of CIK cytotoxic activity on A549 cell line was performed at three effector-to-target cell ratios of 40:1, 20:1, 10:1. Data represent the mean ± SD of six independent experiments.

Peripheral blood mononuclear cells (PBMCs) from 6 individuals with NCSLS were selected for small-scale CIK culture. The subsets of CIK cells were analyzed by flow cytometry (FCM) analysis during 21 days of incubation. The majority of these cells were positive for CD3+ (95.17 ± 3.36%), whereas CD3-CD56+ NK cells were relatively rare (3.0 ± 3.08%) (Figure 1A, 1B, and Figure S1). Among the CD3+ cells, the percentage of CD8+ cells increased from 44.6% to 85.1%. On the contrary, the percentage of CD4+ cells decreased from 47.0% to 12.15%. Furthermore, the total number of CD3+CD56+ NKT cells increased dramatically from 2.75% to 52.5% during CIK cell proliferation (Figure 1C, 1D, 1E, and Figure S1); this subset has been reported to contribute the most to CIK cytotoxic activity [9]. Figure 1F showed that the highest CIK cytotoxic activity on the A549 cell line occurred at day 15, which corresponded to the optimal time of clinical transfusion of CIK cells [12, 16]. Following the analysis of the subsets of CIK cells, the dynamic expression of checkpoint molecules was determined.

### Expression of PD-1 and its ligands, PD-L1 and PD-L2 on CIK cells

**Figure 2:**
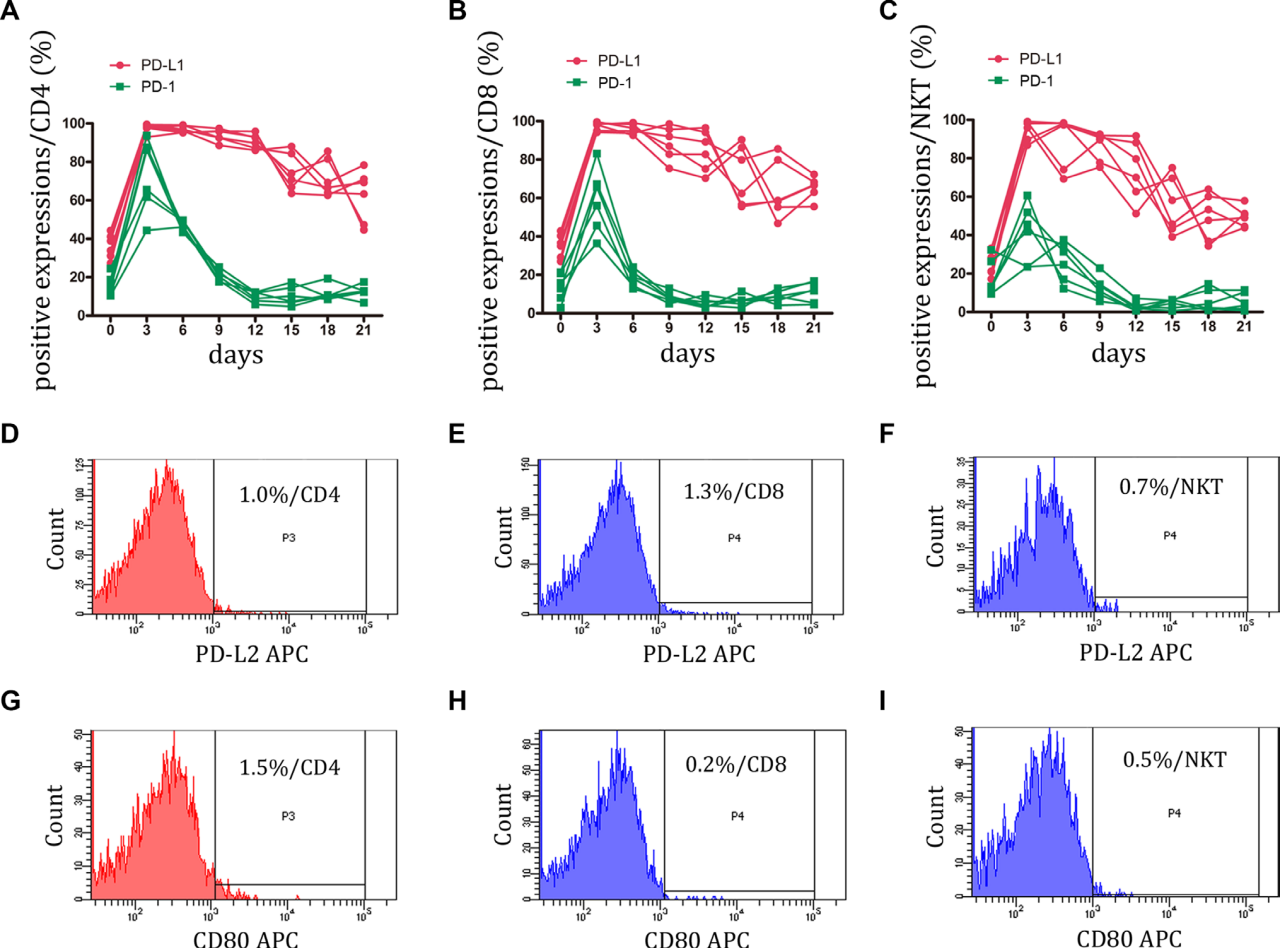
Expression of PD-1 and its ligands, PD-L1 and PD-L2 on CIK cells was detected (**A–C**) During small-scale CIK culture, PD-1 and PD-L1 expressions on CD3+CD4+ T, CD3+CD8+ T and CD3+CD56+ NKT cells were detected by FCM. Data represent six independent experiments. (**D–I**) PD-L2 and B7-1 (CD80) were almost not expressed by CD3+CD4+ T, CD3+CD8+ T and CD3+CD56+ NKT cells. The representative photos were shown.

T cells express PD-1 only after activation when it functions to limit the effector phase of T cell differentiation [17]. In the present study, we investigated whether CIK activation contributes to PD-1 expression. PBMCs were stimulated with anti-CD3 mAb, IL-2, IFN-γ, and IL-1 for 21 days, and PD-1 expression were monitored by FCM. At the early stage of CIK induction, PD-1 expression was increased sharply and reached the maximum value on CD4+, CD8+, and NKT cells. However, after day 3, PD-1 expression gradually decreased to the bottom, particularly in NKT cells. The PD-1 positive percentage was less than 10% after day 12. (Figure 2A, 2B, 2C, and Figure S2)

Of interest, PD-L1 expression on CIK cells remained steadily elevated following the initial rapid increase. At the end of culture (day 21), at least 50% of PD-L1 positive cells were present in each major subset of CIK cells. B7-1 (CD80) has been described as a binding partner for PD-L1 with an intermediate affinity (∼1.7 μM), and this interaction can induce an inhibitory signal of PD-L1 into T cells [17]. Therefore, to determine whether this negative interaction was present between CIK cells, B7-1 expression was assessed on each subset of CIK cells. Throughout the culture, B7-1 was rarely detected on the CIK cells (Figure 2G, 2H, and 2I). The data indicated that CIK cells might be in a special dysfunctional state with PD-L1^high^ PD-1^low^ and that their cytotoxicity might become impaired once they encounter the partner B7-1. Furthermore, the expression of PD-L2, another PD-1 ligand, was undetected on CIK cells (Figure 2D, 2E, and 2F).

### Expression of TIM-3 and CEACAM1 on CIK cells

**Figure 3:**
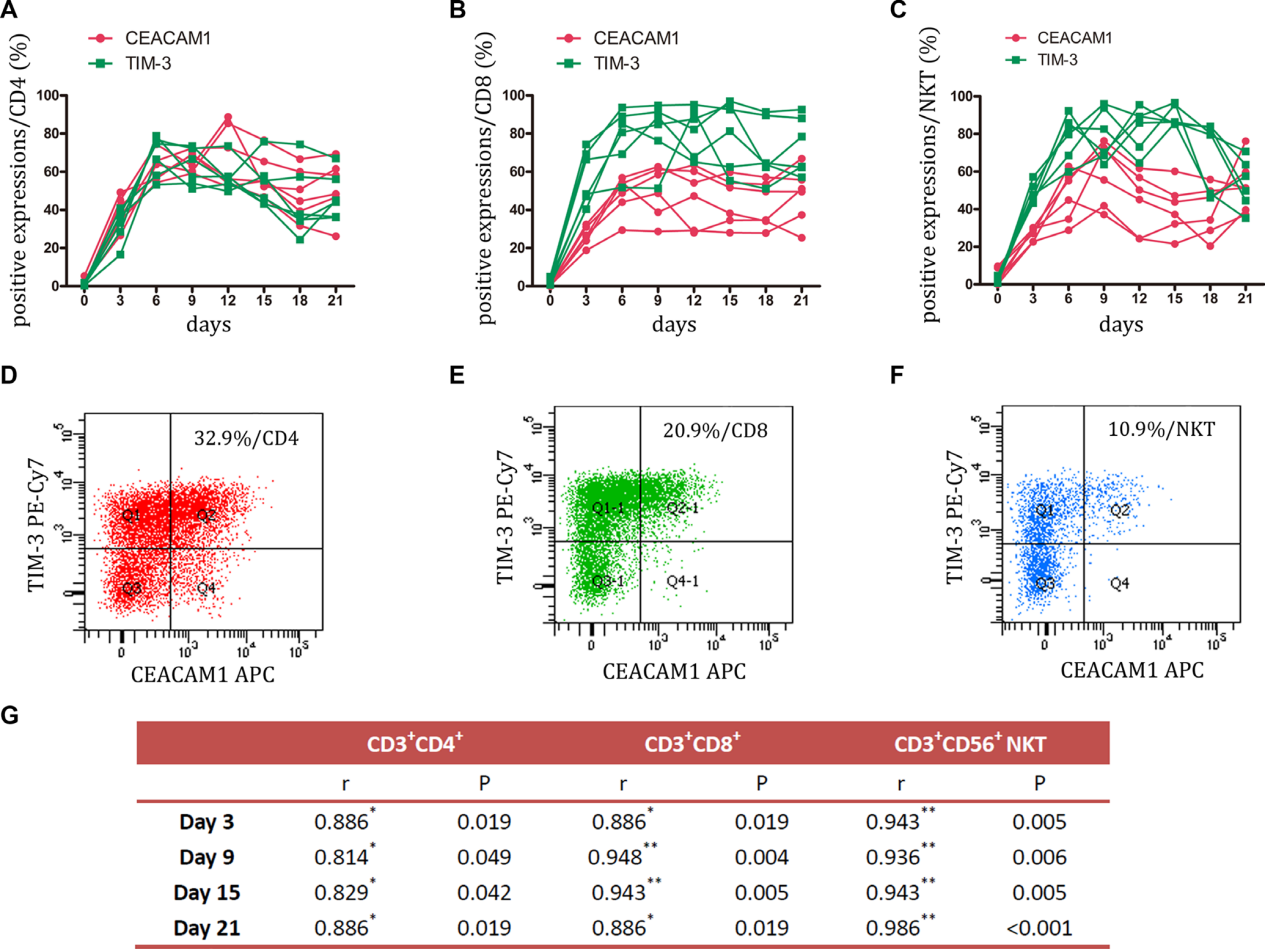
Expression of TIM-3 and CEACAM1 on CIK cells was detected (**A–C**) During small-scale CIK culture, expression of TIM-3 and CEACAM1 on CD3+CD4+ T, CD3+CD8+ T and CD3+CD56+ NKT cells was detected by FCM. Data represent six independent experiments. (**D–F**) FCM was employed to analyze the co-expression of these two inhibitory molecules on CD3+CD4+ T, CD3+CD8+ T and CD3+CD56+ NKT cells. The representative photos were shown. (**G**) Spearman's nonparametric correlation was performed for analyzing the correlation between TIM-3 and CEACAM1 expressions at day 3, 9, 15, 21. *n* = 6; r: correlation coefficient; ^*^P = < 0.05, ^**^P = < 0.01

TIM-3 plays an important role in tumor-induced immune suppression. It has been showed that TIM-3 marks the most suppressed or dysfunctional populations of CD8+T cells in animal models of solid and hematologic malignancies [18, 19]. The present study also found a stable elevation of TIM-3 expression during CIK culture, but its expression was rare on CD4+, CD8+, and NKT cells prior to stimulation. Of interest, CEACAM1, which forms a heterodimeric interaction with TIM-3 [20], was upregulated simultaneously (Figure 3A, 3B, 3C, and Figure S2). Statistical analysis showed that a significantly positive correlation existed between TIM-3 and CEACAM1 expression (Figure 3G). FCM analysis found that these two inhibitory molecules were approximately co-expressed on 30% of CD4+ cells, 20% of CD8+ cells, and 10% of NKT cells (Figure 3D, 3E, and 3F). The co-expression on CD4+ cells was higher than on CD8+ cells or NKT cells. These data suggested that TIM-3 ^high^CEACAM1 ^high^ might also mark the dysfunctional state of CIK cells.

### Expression of BTLA, CTLA-4, TIGIT, and LAG-3 on CIK cells

**Figure 4:**
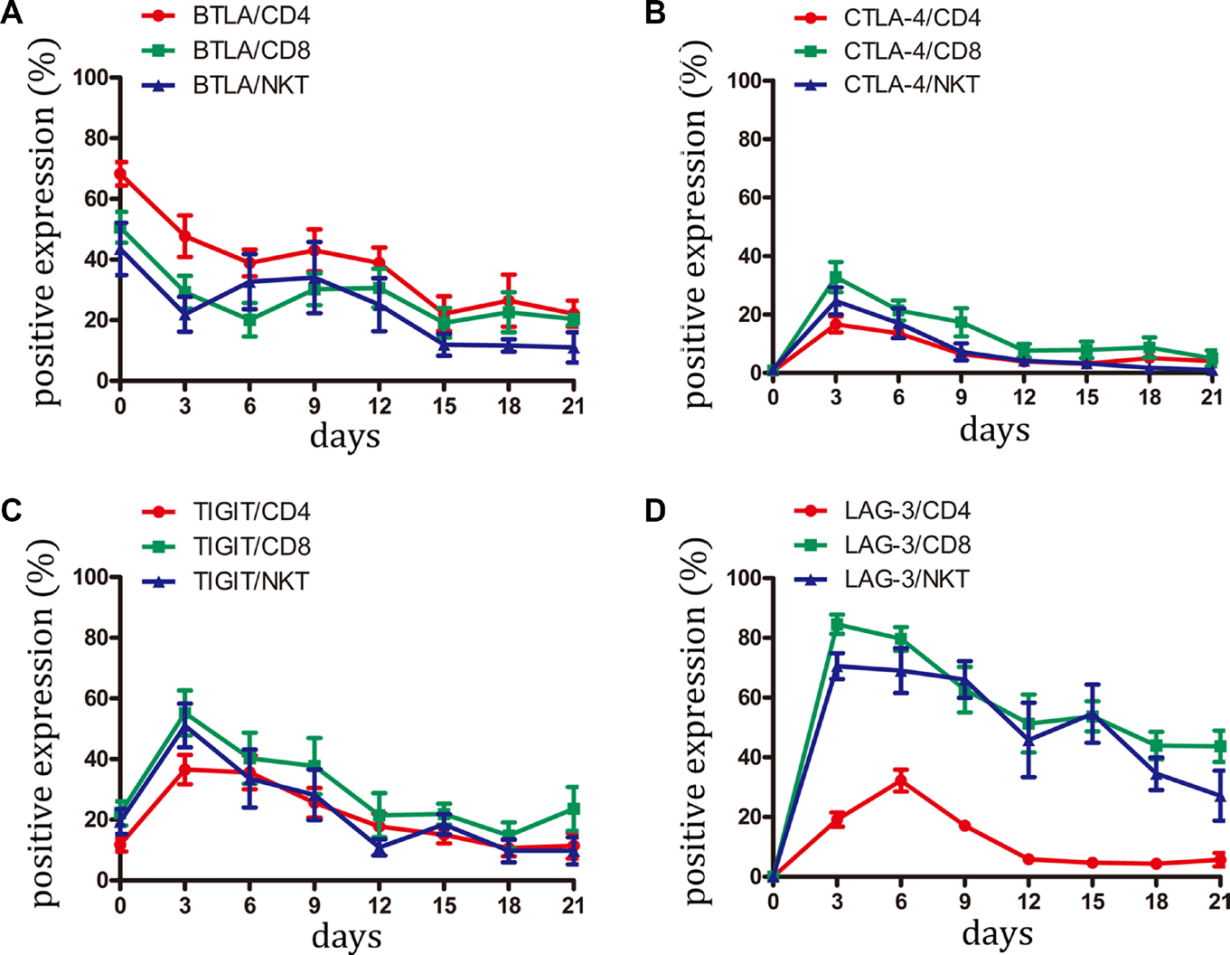
Expression of BTLA, CTLA-4, TIGIT and LAG-3 on CIK cells was detected (**A–D**) During small-scale CIK culture, expression of BTLA, CTLA-4, TIGIT and LAG-3 on CD3+CD4+ T, CD3+CD8+ T and CD3+CD56+ NKT cells was detected by FCM. Data represent the mean ± SD of six independent experiments.

BTLA has been identified as an inhibitory receptor and is mainly expressed by immune cells [21]. BTLA overexpression has been reported in hematological malignancies and melanoma and appears to be associated with impaired tumor-specific T-cell activity, particularly with PD-1 expression [22, 23]. In the current study, BTLA was highly expressed on CD4+, CD8+, and NKT cells from freshly isolated PBMC. However, during CIK activation, BTLA expression gradually decreased from 40%–70% positive cells to 20% positive cells, thus indicating that BTLA might be an unimportant negative receptor on CIK cells (Figure 4A and Figure S3).

CTLA-4 serves as a checkpoint molecule that regulates the amplitude at the early stages of T-cell activation by binding to B7-1 and B7-2 with greater affinity than CD28 [24]. Similar to these data, CTLA-4 expression was only found during the early stage after CIK stimulation. CTLA-4 was almost not detected in the later stage, indicating that it might be not an essential molecule for CIK function (Figure 4B and Figure S3).

TIGIT is a type 1 transmembrane protein containing an immunoreceptor tyrosine-based inhibitory motif (ITIM) in the cytoplasmic tail. Several reports attested that TIGIT negatively regulates T-cell activation [25, 26]. Our study showed that TIGIT was expressed by CD4+, CD8+, and NKT cells from PBMC. Following induction by cytokines, TIGIT expression displayed a similar variation curve to PD-1. After day 15, the percentage of cells expressing TIGIT returned to the level of initial expression (day 0) (Figure 4C and Figure S3).

LAG-3 is a surface molecule that is highly homologous to CD4 in structure and also binds to MHC class II molecules but with high affinity [27]. LAG-3 associates with the TCR:CD3 complex following TCR engagement and negatively regulates signal transduction [28]. The present study demonstrated that LAG-3 was absent in the CD4+, CD8+, and NKT cells derived from peripheral blood. However, LAG-3 expression presented different variations among the subset of CIK cells. Following activation, LAG-3 expression modestly elevated in CD4+ T cells, with the highest value of approximately 30% positive cells. By contrast, LAG-3 expression on CD8+ T and NKT cells sharply ascended to the top at an approximately 80% positive rate. Subsequently, LAG-3 expression gradually decreased. However, at the terminal of the culture, LAG-3 almost vanished on CD4+ T cells but had at least a 30%–40% positive rate on CD8+ T cells and NKT cells (Figure 4D and Figure S3) Given that CD3+CD56+ NKT cells were the dominant cytotoxic cells in the CIK function [9, 12], LAG-3 expression on this cell might indicate a dysfunctional state.

### Checkpoint receptors on CIK cells within large-scale culture system

**Figure 5:**
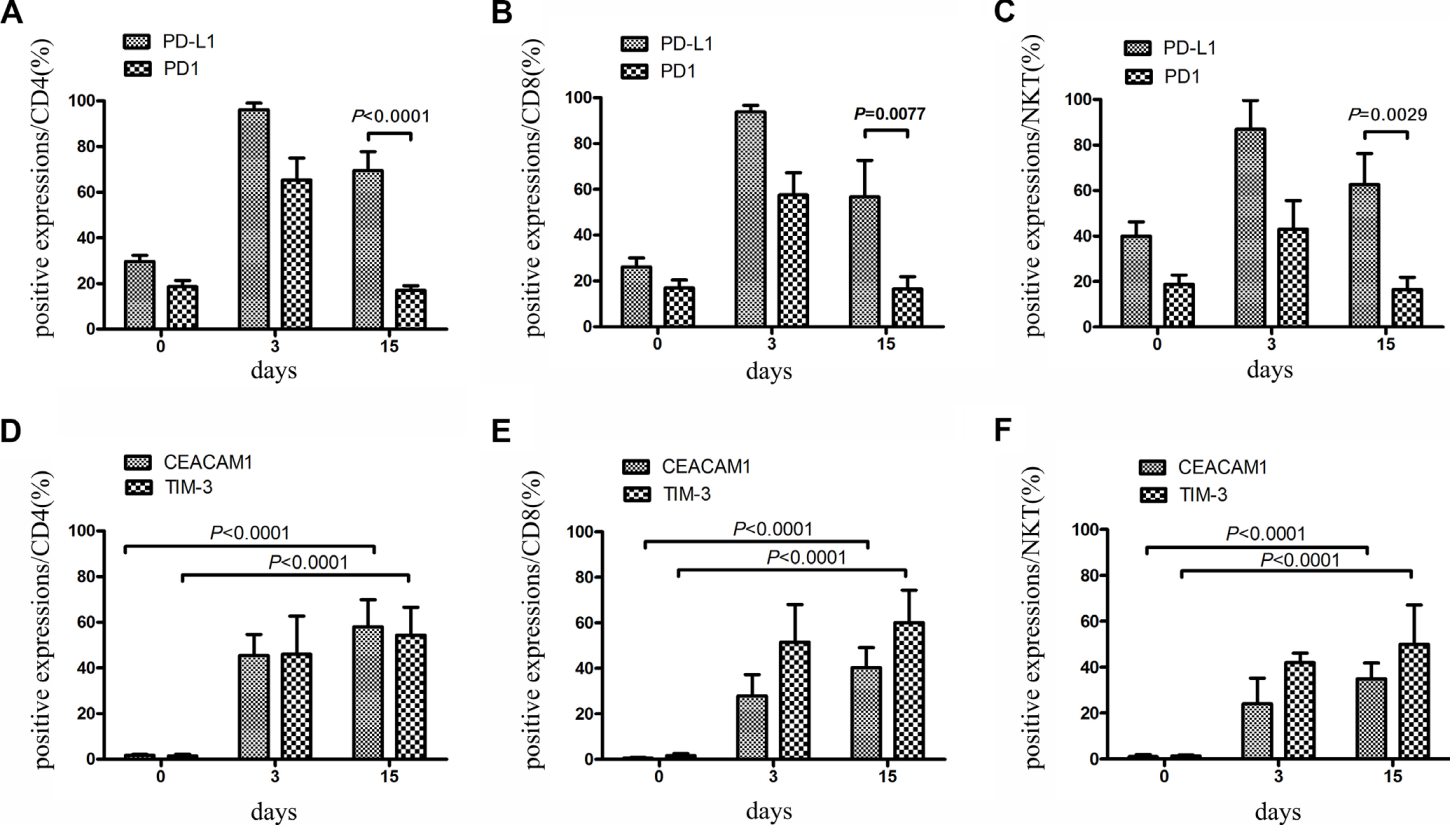
Expression of PD-1 and PD-L1 on CIK cells within large-scale culture system was detected PBMCs from ten individuals with NSCLC were chosen for large-scale CIK culture. (**A–C**) During 15 days of culture, expression of PD-1 and PD-L1 on CD3+ CD4+ T, CD3+CD8+ T and CD3+CD56+ NKT cells was detected at day 0, 3 and 15 by FCM. Data represent the mean ± SD of ten independent experiments. (**D–F**) Expression of TIM-3 and CEACAM1 on CD3+ CD4+ T, CD3+ CD8+ T and CD3+ CD56+ NKT cells was detected at day 0, 3 and 15 by FCM. Data represent the mean ± SD of ten independent experiments.

**Figure 6:**
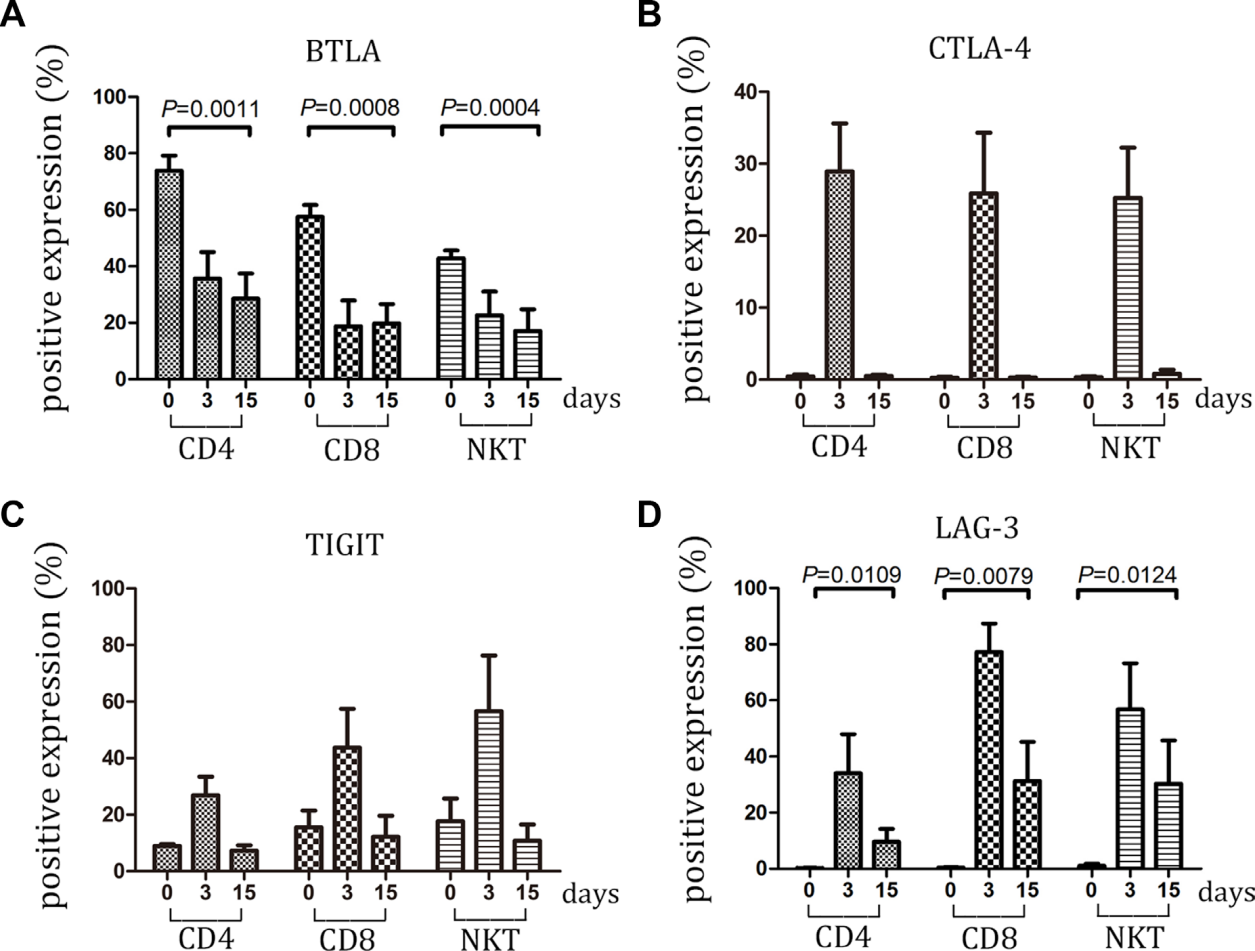
Expression of BTLA, CTLA-4, TIGIT and LAG-3 within large-scale culture system was detected (**A–D**) Expression of BTLA, CTLA-4, TIGIT and LAG-3 on CD3 + CD4 + T, CD3 + CD8 + T and CD3 + CD56 + NKT cells was detected at day 0, 3 and 15 by FCM. Data represent the mean ± SD of ten independent experiments

A total of 10 individuals with NSCLC were chosen to investigate whether similar variations of checkpoint regulators were found on CIK cells within large-scale culture system. In this system, CIK cells were cultured for 15 days followed by clinical transfusion. These cells were detected for the expression of checkpoint proteins at days 0, 3, and 15. The data described in Figures 5 and 6 were nearly in line with those mentioned in small-scale culture systems. Compared with the initial expression on PBMC before cytokine stimulation, the checkpoints on CIK cells might be marked as PD-L1^high^ TIM-3 ^high^CEACAM1 ^high^ LAG-3 ^high^ - BTLA ^low^ TIGIT ^low^ PD-1 ^low^ CTLA-4 ^low^ (Table S1).

## DISCUSSION

Immunotherapy, which has recently received remarkable achievements, has become the fourth most important method for cancer treatment, after surgery, radiotherapy, and chemotherapy [13]. CIK cell, a type of non-specific adoptive immunotherapy, expresses CD3, CD56, and NKG2D antigen and shows MHC-unrestricted cytotoxicity toward neoplastic but not normal cells [9]. We previously reported the results of several retrospective studies in adjuvant immunotherapy with CIK cells for metastatic renal carcinoma, advanced gastric carcinoma, lung cancer, and advanced epithelial ovarian cancer [14–16, 29–31]. Other groups also reported the therapeutic results of CIK immunotherapy for different cancers [13]. Nevertheless, CIKs therapy only benefits a small portion of patients. Thus, we considered there might be some inhibitory mechanisms that restrain CIK cytotoxicity, such as checkpoint regulators. To our knowledge, the present study is the first demonstration that is related to checkpoint molecules on CIK cells.

In a previous study on viral chronic infection (2007), T cells that are reactive to viral antigens lose their ability to kill virus-infected cells upon chronic antigen exposure. Dysfunctional T cells, which are also called exhausted cells, are characterized by an upregulation of a panel of receptors with negative regulatory functions such as PD-1, CTLA4, TIM3, LAG3, and CD244 (2B4) [31]. To some extent, a CIK cell culture is similar to a viral chronic infection because the CIK cells have been activated and expanded in a long-term culture (15 days) *in vitro*. In the present study, dynamic changes were observed in the inhibitory phenotypes. The majority of these checkpoint molecules, except BTLA, were sharply elevated during the early stage of CIK cell culture. However, the subsequent expressions of these inhibitory receptors showed different results.

PD-1 and its ligands, PD-L1 and PD-L2, have gained considerable interest because their therapeutic targeting has shown remarkable success in clinical trials [32]. The induction of PD-1 and PD-L1 expression on human T cells has been previously described under TCR stimulation (anti-CD3/CD28 mAb stimulation) or γc cytokine-mediated immune activation, such as IL-2, IL- 7, IL-15, and IL-21 [33]. In the present study, PD-1 and PD- L1 expressions on CIK cells also increased sharply at the early stage because of stimulation by anti-CD3 mAb, IL-2, IFN-γ, and IL-1. Nevertheless, the subsequent expression of PD-1 decreased gradually towards the initial level. The mechanism of this phenomenon is still unclear.

To our surprise, PD-L1 remained stably upregulated during CIK culture compared with PD-1, thus indicating that PD-L1 could act as an inhibitory molecule on CIK cells instead of PD-1. B7-1:PD-L1 interactions delivers a functionally significant inhibitory signal into the T cells [17]. B7-1(CD80), which exists on the membrane of activated antigen presenting cells (APC), has been identified as a binding partner for PD-L1 with an affinity (∼1.7 μM) between the affinities of B7-1 for CD28 (4 μM) and CTLA-4 (0.2 μM) [17]. Given B7-1 and PD-1 expressions were low on the CIK cells (Day 15), the interaction between CIK cells via PD-L1:PD-1 or PD-L1:B7-1 may not be important. However, when CIK cells were transfused into the patient's blood, B7-1 on the APC surface *in vivo* could engage with PD-L1 on the CIK surface, thus impairing CIK cytotoxicity to some degree.

PD-L2 (B7-DC) expression was reported to be largely restricted to dendritic cells (DCs) and activated macrophages. In the present study, PD-L2 expression was not observed in CIK cells. However, one study found PD-L2 expression in mouse T cells upon T-cell immune response [34].

TIM-3 was initially identified as a specific marker of fully differentiated IFN-γ producing CD4^+^ T helper 1 (Th1) and CD8 cytotoxic (Tc1) cells [35]. In addition to T cells, TIM-3 is also highly expressed on monocytes, macrophages, and DCs [36]. The current study first reported that TIM-3 expression was upregulated on CIK cells. Although TIM-3 was rarely expressed on CD4+, CD8+, and NKT cells from PBMC, all subsets of CIK cells strongly expressed TIM-3. Moreover, TIM- 3 expression remained at a high level during long-term culture. Similarly, a drastic elevation of TIM-3 expression has been reported on human naïve CD4+ T cells activated with plate-bound anti-CD3/anti-CD28 for seven days [37]. Furthermore, the regulation mechanism of Tim-3 expression is involved in Th1-specific transcription factor T-bet in mice [38].

Some reports demonstrated that binding of Gal- 9 to TIM-3 causes an inhibitory signal, resulting in apoptosis of Th1 cells and cytotoxic CD8 T cells *in vitro* [39, 40]. Galectin-9 (Gal-9), acted as one of the TIM-3 ligands, is widely distributed in tissues involved in the immune system, i.e. spleen, thymus and peripheral blood lymphocytes, and in tissues of endodermal origin, i.e. liver, intestine, stomach and lung [41, 42]. Therefore, when CIK cells encountered Gal-9 *in vivo*, the cytotoxicity of CIK cells might be attenuated.

CEACAM1 is expressed on a variety of cells and has multiple biological functions, especially cell to cell adhesion [43]. It also acts as an inhibitory molecule that negatively regulates cytotoxic T cell proliferation via agonist monoclonal antibodies [44], and inhibits NK cell mediated killing by its homotypic interaction [45]. Of interest, we also observed the high expression of CEACAM1 with the increase of TIM-3 and co-expression of the two proteins on the CIK cells. This observation was in agreement with a recent study by Huang [20], who showed that the presence of CEACAM1 endowed TIM-3 inhibitory functions. CEACAM1 facilitates the maturation and surface expression of TIM-3 by forming a heterodimeric interaction, and the co-blockade of CEACAM1 and TIM-3 enhanced the anti-tumor immune response [20]. The high expression of TIM- 3 and CEACAM1 indicated that CIK cells might be in a dysfunctional state. When these cells encountered galectin-9 (Gal-9), a widely expressed soluble molecule acted as one of the TIM-3 ligands, *in vivo*, the cytotoxicity of CIK cells would be attenuated.

CTLA-4 (CD152) has been actively studied. It primarily regulates the amplitude of the early stages of T-cell activation by outcompeting CD28, which is an important co-stimulatory molecule that is constitutively expressed on T cells, in binding CD80 and CD86. CTLA-4 is not expressed by naïve CD4+ or CD8+ T cells; however, it is contained intracellularly and induced trafficking to the cell surface upon TCR engagement where it actively delivers inhibitory signals into the T cell [46]. Similar to these data, CTLA-4 expressions in CD4+, CD8+, and NKT cells were also detected at an early stage in the context of CIK culture. But afterwards, the expressions faded away gradually, thus suggesting that CTLA-4 might not be essential for final CIK function.

BTLA was identified as another inhibitory molecule that has structural similarities with CTLA-4 and PD-1 [21]. BTLA expression has been reported to be limited in lymphoid tissues, highest in B cells, and significant in αβ and γδ T cells, mature DCs, and macrophages [21]. The present study first demonstrated that isolated NKT cells from PBMC highly expressed BTLA constitutively similar to CD4+ or CD8+ T. Upon stimulation of CIK culture, BTLA was continuously downregulated to the lowest level on fully activated CIK cells; this phenomenon distinguished BTLA from other checkpoint proteins. This data was in line with the report related to the kinetic alteration of BTLA expression on naïve T cells in the activation process [47, 48]. Similar to CTLA-4, BTLA might not be critical for CIK final attack.

Some findings have placed TIGIT as a vital immunomodulator protein because it is able to control the activities of both NK and T cells [25, 49]. TIGIT has been reported to be expressed by freshly isolated NK, NKT, CD8+, and CD4+ T cells from peripheral blood [25, 49]. This result was also found in the present study. By adding cytokines to the CIK culture, TIGIT expression dramatically increased to the maximum value and then decreased gradually to the initial level of expression (day 0), similar to PD-1.

LAG-3, another inhibitory molecule, is expressed on activated T cells, NK cells, B cells and plasmacytoid DCs, and plays an important role in the negative regulation of T-cell proliferation via binding to the MHC class II with high affinity [50, 51]. Nevertheless, there was no expression of LAG-3 protein on CD4+, CD8+, and NKT cells from freshly isolated PBMC, similar to TIM3, CEACAM1, and CTLA4. After being induced by the cytokines of CIK culture, LAG-3 expression also underwent a variation from high to low. However, a significant difference of LAG-3 levels was observed between CD4+, CD8+, and NKT cells. This data indicated that the LAG-3 modulation of CD4+ T cells might have a distinct mechanism which still remained unknown. At day 15, the level of LAG-3 expression on CD8+ T and NKT cells was significantly higher than the initial level.

In summary, this study provides evidence that immune checkpoint regulators on CIK cells showed dynamic expressions during the culture. This variation occurred in both small- and large-scale cultures. Most of these molecules were induced upon CIK cell activation. However, during long-term culture, their expression showed significant diversity. Of note, at day 15, CIK cells might be partly exhausted, which is characterized by high expression of PD-L1, LAG-3, TIM-3, and CEACAM-1 and low expression of TIGIT, BTLA, PD-1, and CTLA- 4 compared with their initial expression (Table S1). It remains to be demonstrated whether these receptors could induce CIK effector response terminated/constrained by feedback inhibition when being engaged with their ligands *in vivo* or *in vitro* (e.g., CD80 or Gal-9). Furthermore, implementing combined treatment on CIK cells before transfusion via antibodies that target PD-L1, LAG-3, TIM- 3, and CEACAM-1 might improve the efficiency of CIK therapy for individuals with NSCLC.

## MATERIALS AND METHODS

### Patients

A total of 16 patients with NSCLC were enrolled, including 3 patients with squamous carcinoma and 13 patients with adenocarcinoma but free of congestive heart failure, severe coronary artery disease, cardiac arrhythmias, HIV infection, chronic active hepatitis, and concomitant corticosteroid therapy. The clinical characteristics of the patients were summarized in Table 1. This study was approved by the State Food and Drug Administration of China (2006L01023) and by the Ethical Committee of Cancer Hospital of Tianjin Medical University, Tianjin, China, according to the guidelines of the Declaration of Helsinki. Informed consent was obtained from all subjects before their entry into the study.

**Table 1:** Basic information of patient samples

Clinico-pathologic factors	Variable	Number
**Pathological subtypes**	Adenocarcinoma	13(16)
	Squamous cell lung cancer	3(16)
**Age**	40–60	8(16)
	≥ 60	8(16)
**Gender**	Male	9(16)
	Female	7(16)
**Stage**	I	4(16)
	II	0(16)
	III	3(16)
	IV	9(16)
**Lymphatic invasion before suegery**	presence	9(16)
	absence	7(16)
**Surgery**	yes	12(16)
	no	4(16)
**Tumor bearing**	presence	11(16)
	absence	5(16)

### CIK cells preparation

CIK cells were prepared as described in our previous studies [14, 16]. Briefly, in large-scale culture system, 40 ml PBMCs were collected from the patients with NSCLC by using a Cobe Spectra Apheresis System (CaridianBCT). The PBMCs were then cultured in AIM-V medium (Invitrogen) containing 50 ng/mL anti-CD3 antibody (e-Bioscience), 100 U/mL recombinant human IL-1α (e-Bioscience), and 1,000 U/mL IFN-γ (PeproTech), at 37°C with 5% CO_2_ for 24 hours. Thereafter, 300 U/mL recombinant human IL-2 (rhIL-2; Proleukin) was added into the media. The medium was replaced by fresh IL-2, and the IFN-γ-containing medium was replaced every 5 days. At day 15, CIK cells were harvested and transfused into NSCLC patients. At the same time, CIK cells were analyzed for phenotype and cytotoxicity. All products were free of bacterial, mycoplasma, or fungal contamination and contained < 5 Eu endotoxin. In small-scale culture system, 4 ml PBMC was used for CIK proliferation for 21 days.

### Detecting the subsets of CIK cells

Subsets of untreated PBMCs and autologous CIK cells from 16 patients with NSCLC were detected by multiple-color fluorescence as described in our previous studies [14, 16]. Briefly, according to the routine method, 5 × 10^5^ CIK cells were resuspended in 20 μl of PBS containing 2% newborn calf serum and 1% sodium azide and then labeled with anti-CD3-FITC/anti-CD56-RPE (Dako), CD3-Percp, CD4-FITC (fluorescein isothiocyanate), CD8-RPE (BD Bioscience). The cell population was analyzed using flow cytometry (FCM) (BD Aria).

### Detecting the checkpoint phenotype on CIK cells

The expression profile of checkpoint receptors on CIK cells was analyzed every three days throughout the culture by using flow cytometry (BD Aria). These receptors expression were assessed using anti-human antibodies, including PD-1, PD-L1, PD-L2, CD28, B7-1(CD80), CTLA4, TIM3, CEACAM-1, TIGIT, LAG3, and BTLA. All mAbs were purchased from BioLegend Company unless otherwise indicated.

### Detecting cytotoxicity of CIK cells

The cytotoxicity of CIK cells from 6 patients was detected, as described in our previous studies [14, 16]. Briefly, the target cell used for this assay was A549, which is the cell line of lung adenocarcinoma. The cell lines were obtained from the American Type Culture Collection. Target cells (1 × 10^5^ cells/mL) were incubated for 4 hours in triplicate sets with effector cells (CIK cells) at effector-to-target cell ratios of 40:1, 20:1, and 10:1. At the end of incubation, 50 μl culture supernatant was transferred to a new flat 96-well plate and incubated with 50 μl lactate dehydrogenase (LDH) substrate mixture (for the detection of LDH released upon cell lysis) at room temperature for 30 minutes in the dark. Thereafter, 50 μl stop solution was added to each well. Absorbance was measured at 490 nm by using a 96-well plate reader. Killing efficiency was calculated as follows: %killing efficiency = [(experimental counts − effector spontaneous counts − target spontaneous counts)/(target maximal counts − target spontaneous counts)] × 100.

### Statistical methods

Each experiment was repeated at least three times. All data were summarized and represented as mean ± SD. Spearman's nonparametric correlation and two-tailed student's *T* tests was used for statistical analyses. Statistical analyses were performed with SPSS Statistics 22.0 (Armonk, NY, United States) and Graph Pad Prism v.5 (La Jolla, CA, USA). *P* < 0.05 was considered statistically significant.

## SUPPLEMENTARY MATERIALS


