# Supplementary material for: Profiling the dynamic expression of checkpoint molecules on cytokine-induced killer cells from non-small-cell lung cancer patients
Source: Oncotarget. 2016 Jun 7;7(28):43604–15. doi: 10.18632/oncotarget.9871 (PMC5190047; doi:10.18632/oncotarget.9871)
Supplement: Supplementary file 1 [file oncotarget-07-43604-s001.pdf]

Profiling the dynamic expression of checkpoint molecules on cytokine-induced killer cells from non-small-cell lung cancer patients

Supplementary Materials

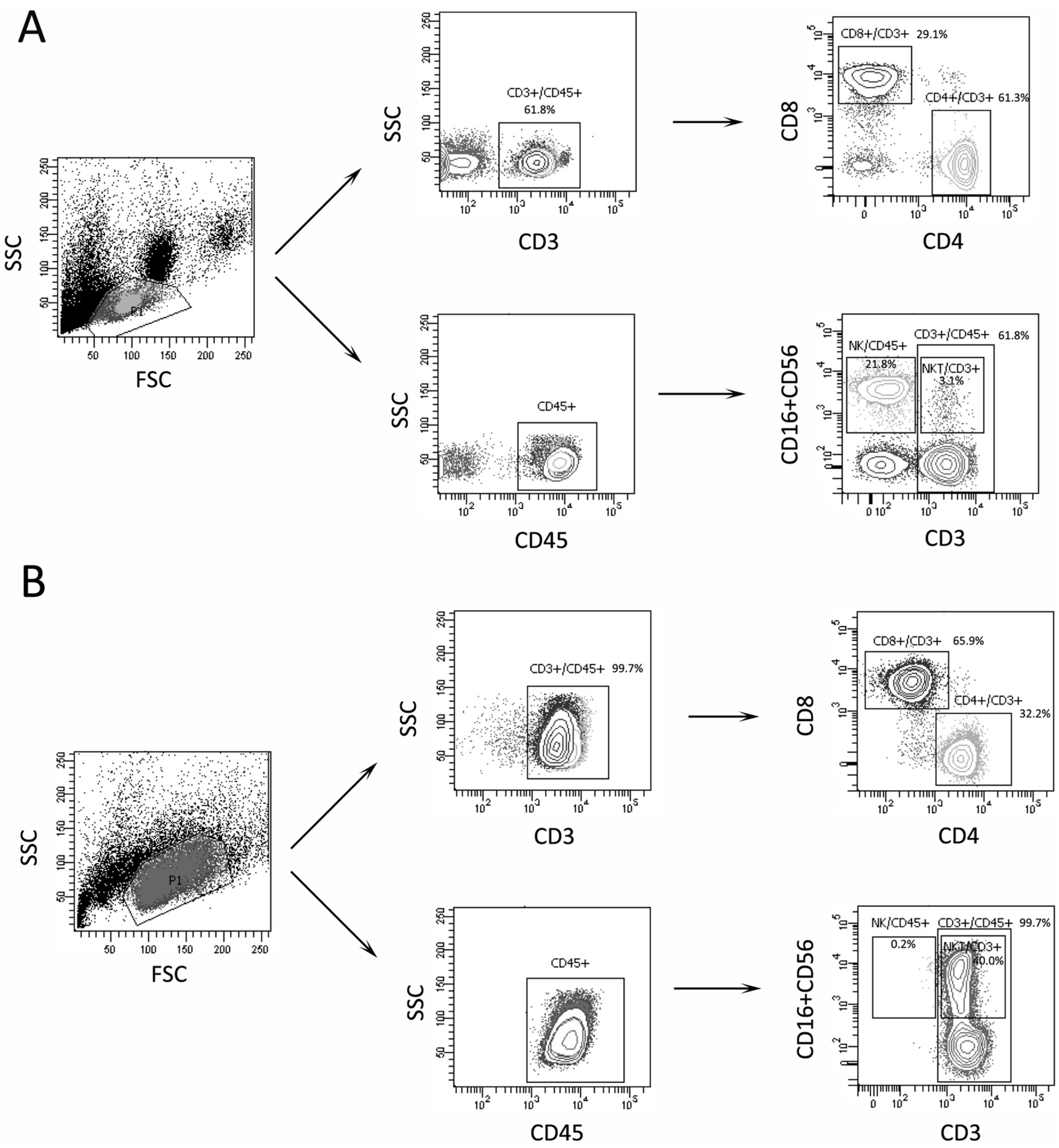

Supplementary Figure S1: Subsets of CIK cells were detected in small-scale culture system, related to Figure 1. PBMCs from patients with NCSLS were selected for small-scale CIK culture. During incubation, the subsets of CIK cells were studied by FCM analysis. (**A–B**) The dynamic percentage of subsets of CIK cells (CD3-CD56+ NK cells, CD3+ T) in CD45+ immune cells was shown. The dynamic percentage of subsets of CIK cells (CD3+CD4+ T, CD3+CD8+ T, CD3+CD56+ NKT cells) in CD3+ immune cells was shown. Typical flow data at day 0 (A) and day 15 (B) were represented.

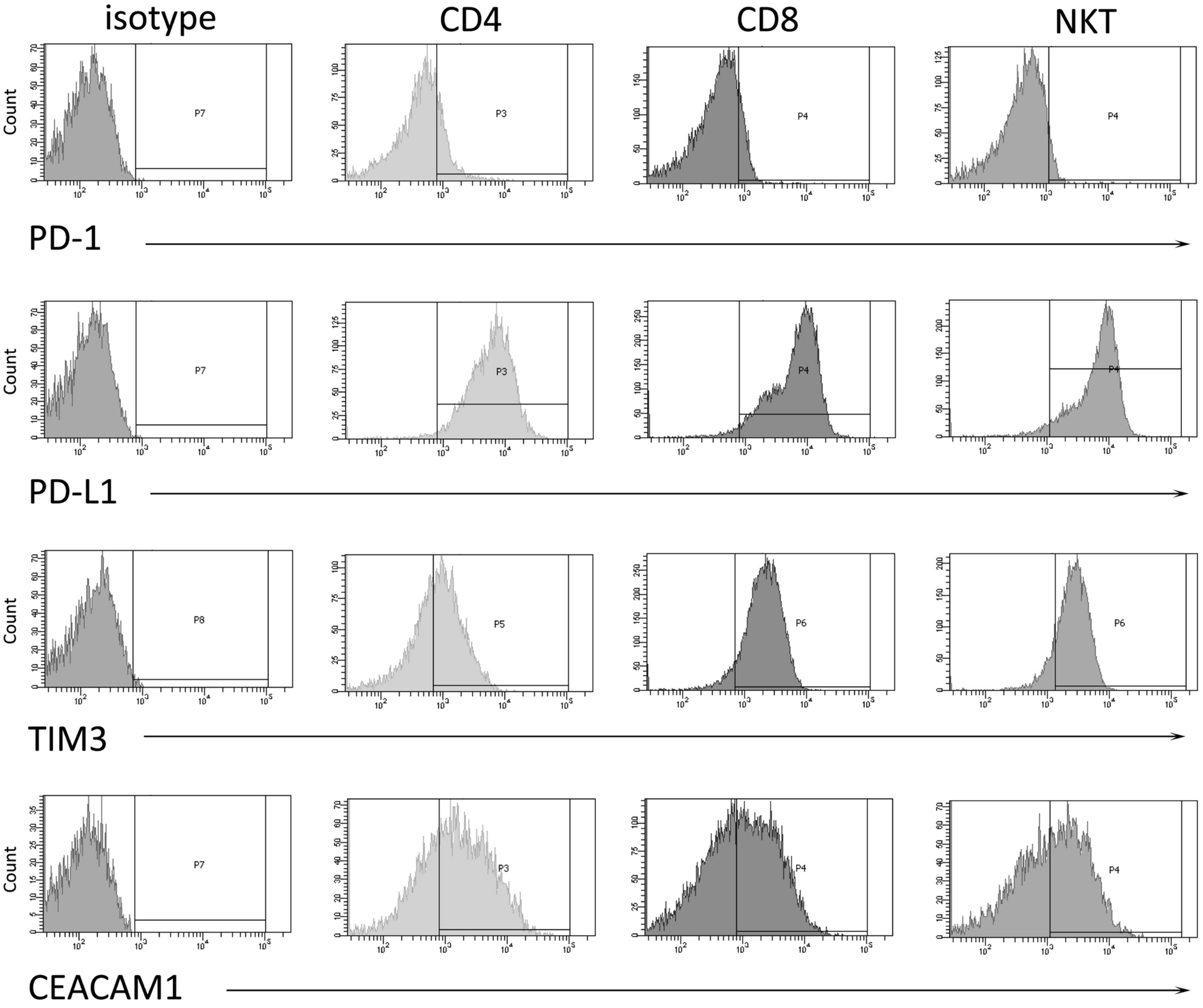

Supplementary Figure S2: Expression of PD-1, PD-L1, TIM-3 and CEACAM1 on CIK cells was detected, related to Figure 2 and Figure 3. During small-scale CIK culture, PD-1, PD-L1, TIM-3 and CEACAM1 expressions on CD3+CD4+ T, CD3+CD8+ T and CD3+CD56+ NKT cells were detected by FCM. Typical flow data at day 15 were represented.

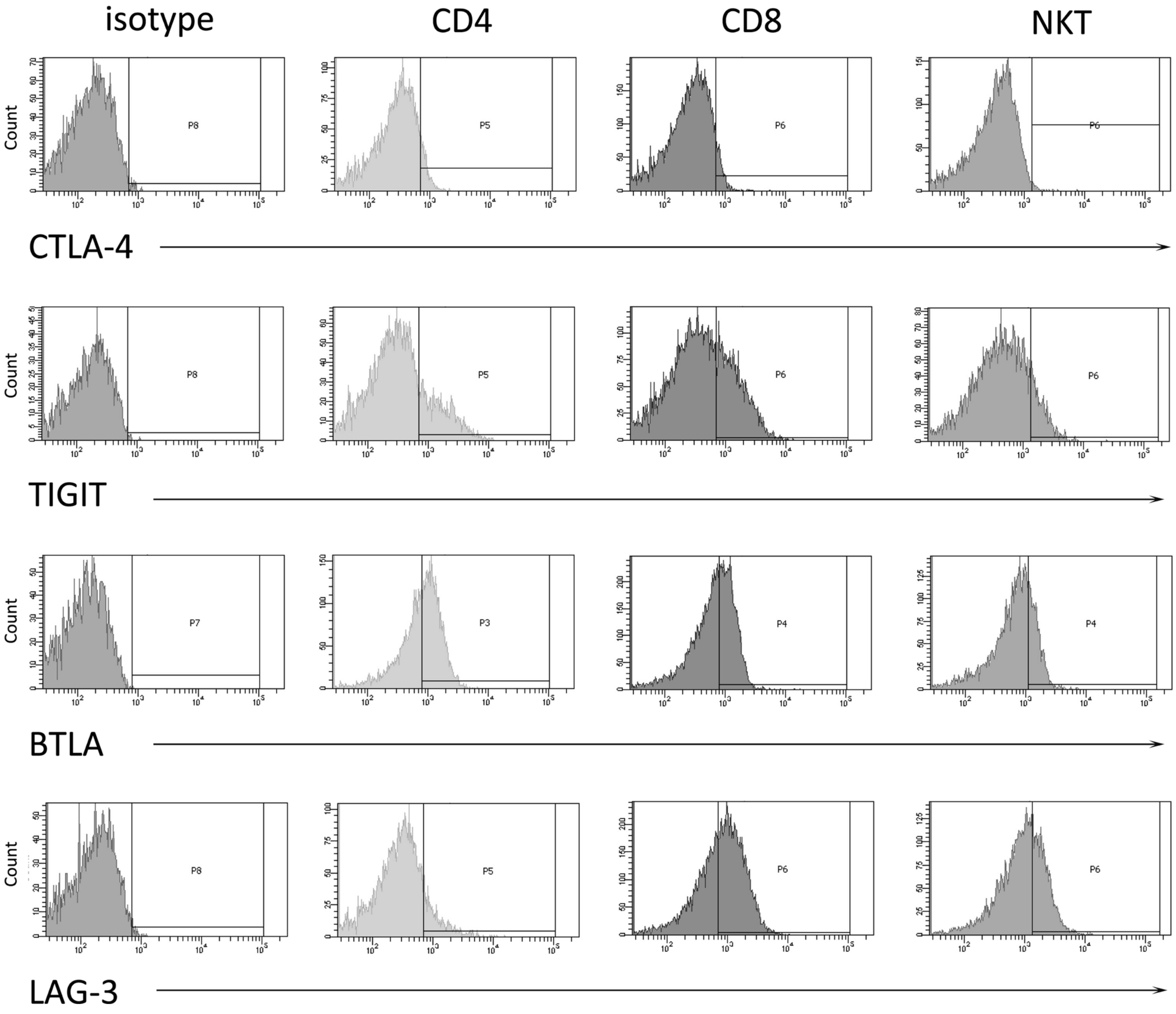

Supplementary Figure S3: Expression of CTLA-4, TIGIT, BTLA and LAG-3 on CIK cells was detected, related to Figure4. During small-scale CIK culture, expression of CTLA-4, TIGIT, BTLA and LAG-3 on CD3+CD4+ T, CD3+CD8+ T and CD3+CD56+ NKT cells was detected by FCM. Typical flow data at day 15 were represented.

Supplementary Table S1: Compared with their initial expression (day 0), the expression of checkpoint receptors on subsets of CIK cells (day 15) were represented, related to Figure 5 and Figure 6

CD3+CD4+ Cells				
High expression	PD-L1	LAG-3	TIM-3	CEACAM-1
Low expression	PD-1	TIGIT	BTLA	CTLA-4
**CD3+CD8+ Cells**				
High expression	PD-L1	LAG-3	TIM-3	CEACAM-1
Low expression	PD-1	TIGIT	BTLA	CTLA-4
**CD3+CD56+NKT Cells**				
High expression	PD-L1	LAG-3	TIM-3	CEACAM-1
Low expression	PD-1	TIGIT	BTLA	CTLA-4
